# *PLOS Computational Biology* 2018 Reviewer and Editorial Board Thank You

**DOI:** 10.1371/journal.pcbi.1006885

**Published:** 2019-02-27

**Fig 1:**
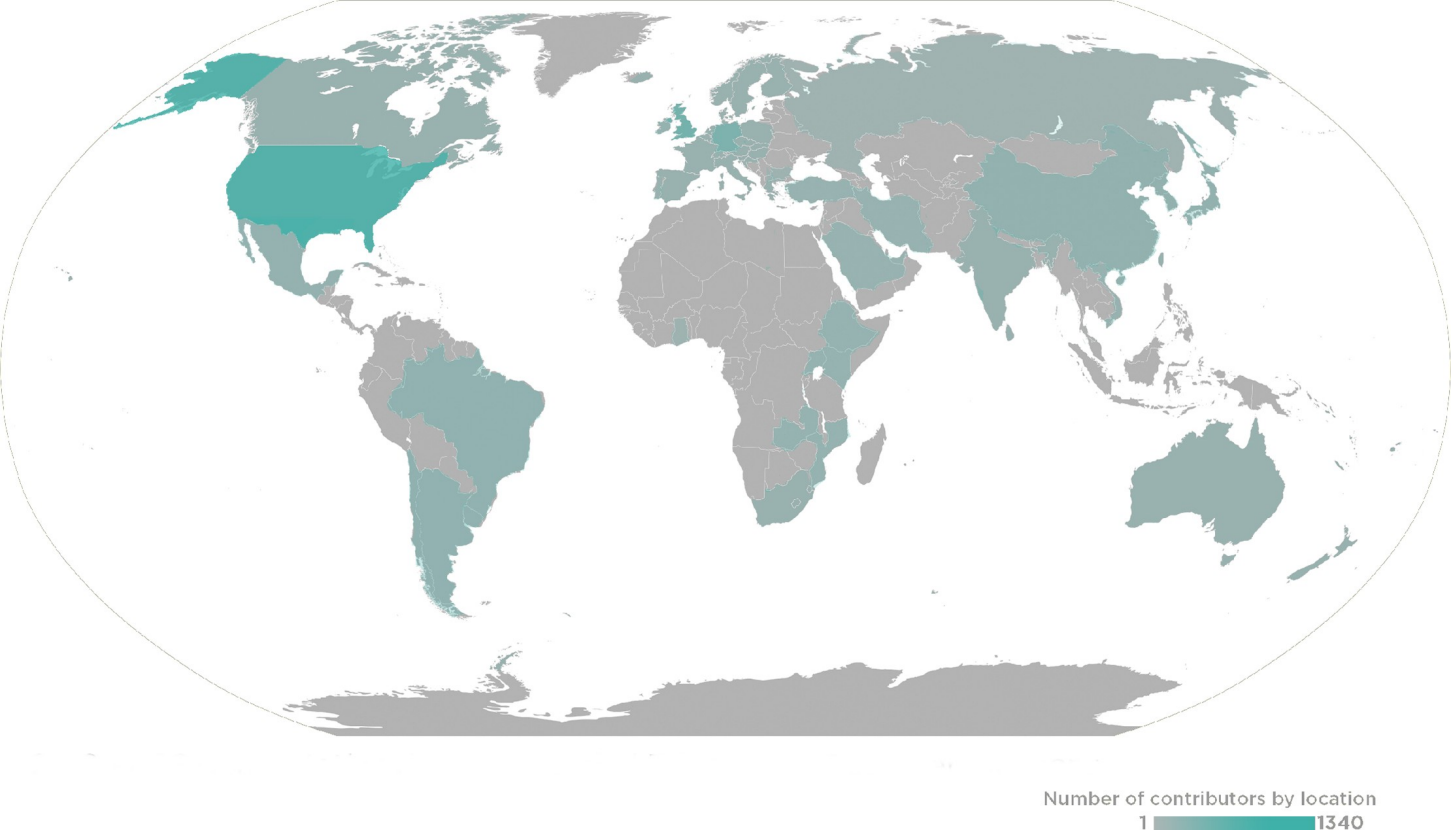
2018 *PLOS Computational Biology* Global Editor and Reviewer Locations.

PLOS and the *PLOS Computational Biology* team want to sincerely thank all of our Editorial Board Members, Guest Editors, and Reviewers for the journal in 2018. Your contributions of time and expertise support your research community, advance scientific progress, and continue to make *PLOS Computational Biology* a leader in its field. This past year, *PLOS Computational Biology* received the assistance of 154 Editorial Board members, 304 Guest Editors, and 2,504 Reviewers, who handled 1,806 manuscripts that resulted in 573 publications (Fig 1).

We’re deeply grateful to all of our volunteers whose dedicated efforts support *PLOS Computational Biology* and Open Science. Thank you all for your work!
